# Supplementary material for: Real-World Evaluation of Calcimimetics for the Treatment of Secondary Hyperparathyroidism in Chronic Kidney Disease, in an Italian Clinical Setting
Source: Healthcare (Basel). 2022 Apr 11;10(4):709. doi: 10.3390/healthcare10040709 (PMC9025876; doi:10.3390/healthcare10040709)
Supplement: Supplementary file 1 [file healthcare-10-00709-s001.zip › healthcare-1628983-supplementary.pdf]

Supplementary Table S1. Baseline characteristics of Cinacalcet- and Etelcalcetide-treated cohorts, Post PSM

	Cinacalcet-treated cohort N=432	Etelcalcetide-treated cohort N=432	P-value
Age at the index-date, mean (SD)	63.7 (15.0)	64.4 (14.5)	0.530
Male, n (%)	270 (62.5)	276 (63.9)	0.672
CCI, mean (SD)	1.1 (1.1)	1.1 (1.1)	0.877
Co-morbidities/clinical manifestations			
Diabetes mellitus, n (%)	99 (22.9)	93 (21.5)	0.623
Hypertension, n (%)	336 (77.8)	323 (74.8)	0.299
CVD, n (%)	60 (13.9)	56 (13.0)	0.690
Fractures, n (%)	4 (0.9)	4 (0.9)	1.000
Hypercalcemia, n (%)	0 (0.0)	0 (0.0)	-
Parathyroidectomy, n (%)	NI	0 (0.0)	0.317
Malignat tumors, n (%)	NI	5 (1.2)	0.477
Hypocalcemia, n (%)	0 (0.0)	0 (0.0)	-
Treatments			
ACE inhibitors, n (%)	129 (29.9)	96 (22.2)	<0.050
Sartans, n (%)	90 (20.8)	103 (23.8)	0.288
Beta Blockers, n (%)	233 (53.9)	229 (53.0)	0.785
Calcium supplementation, n (%)	43 (10.0)	40 (9.3)	0.729
Phosphorus binders, n (%)	277 (64.1)	292 (67.6)	0.282
Vitamin D in active form, n (%)	110 (25.5)	109 (25.2)	0.938
Vitamin D in inactive form, n (%)	270 (62.5)	254 (58.8)	0.265
ESA, n (%)	339 (78.5)	351 (81.3)	0.309
Prednisolone and other steroids, n (%)	54 (12.5)	53 (12.3)	0.918
Anti-osteoporotic medications, n (%)	6 (1.4)	5 (1.2)	0.762

NI (not issuable).

Supplementary Table S2. Baseline characteristics of Cinacalcet- and Etelcalcetide-treated naive patients, Post PSM (1:2)

	Cinacalcet-treated cohort N=552	Etelcalcetide-treated cohort N=276	P-value
Age at the index-date, mean (SD)	65.2 (14.3)	65.4 (14.7)	0.821
Male, n (%)	340 (61.6)	177 (64.1)	0.477
CCI, mean (SD)	1.2 (1.2)	1.1 (1.1)	0.751
Co-morbidities/clinical manifestations			
Diabetes mellitus, n (%)	165 (29.9)	71 (25.7)	0.211
Hypertension, n (%)	432 (78.3)	213 (77.2)	0.722
CVD, n (%)	86 (15.6)	41 (14.9)	0.785
Fractures, n (%)	8 (1.4)	NI	0.668
Hypercalcemia, n (%)	NI	0 (0.0)	0.479
Parathyroidectomy, n (%)	NI	0 (0.0)	0.479
Malignat tumors, n (%)	9 (1.6)	NI	0.283
Hypocalcemia, n (%)	0 (0.0)	0 (0.0)	-

Treatments			
ACE inhibitors, n (%)	176 (31.9)	72 (26.1)	0.086
Sartans, n (%)	122 (22.1)	67 (24.3)	0.482
Beta Blockers, n (%)	264 (47.8)	148 (53.6)	0.116
Calcium supplementation, n (%)	50 (9.1)	22 (8.0)	0.601
Phosphorus binders, n (%)	323 (58.5)	154 (55.8)	0.456
Vitamin D in active form, n (%)	166 (30.1)	75 (27.2)	0.387
Vitamin D in inactive form, n (%)	327 (59.2)	162 (58.7)	0.881
ESA, n (%)	455 (82.4)	233 (84.4)	0.471
Prednisolone and other steroids, n (%)	68 (12.3)	36 (13.0)	0.767
Anti-osteoporotic medications, n (%)	8 (1.4)	4 (1.4)	1.000

NI (not issuable).
